# Novel Therapeutic Method for Unresectable Pancreatic Cancer—The Impact of the Long-Term Research in Therapeutic Effect of High-Intensity Focused Ultrasound (HIFU) Therapy

**DOI:** 10.3390/curroncol28060409

**Published:** 2021-11-20

**Authors:** Atsushi Sofuni, Yasutsugu Asai, Takayoshi Tsuchiya, Kentaro Ishii, Reina Tanaka, Ryosuke Tonozuka, Mitsuyoshi Honjo, Shuntaro Mukai, Kazumasa Nagai, Kenjiro Yamamoto, Yukitoshi Matsunami, Takashi Kurosawa, Hiroyuki Kojima, Toshihiro Homma, Hirohito Minami, Ryosuke Nakatsubo, Noriyuki Hirakawa, Hideaki Miyazawa, Yuichi Nagakawa, Akihiko Tsuchida, Takao Itoi

**Affiliations:** 1 Department of Gastroenterology and Hepatology, Tokyo Medical University, 6-7-1 Nishishinjuku Shinjuku-ku, Tokyo 160-0023, Japan; yacchan85@yahoo.co.jp (Y.A.); tsuchiya623@mac.com (T.T.); ishiken@tokyo-med.ac.jp (K.I.); onakasuicyatta@yahoo.co.jp (R.T.); tonozuka1978@gmail.com (R.T.); 3244honjo@gmail.com (M.H.); maezora1031@yahoo.co.jp (S.M.); kazu4439@gmail.com (K.N.); kenjirojiro5544@yahoo.co.jp (K.Y.); yukitoshimatsunami1228@yahoo.co.jp (Y.M.); takasikurosawa716@yahoo.co.jp (T.K.); ggswh149@yahoo.co.jp (H.K.); ssautuman@gmail.com (T.H.); oti.hor.ih.m@gmail.com (H.M.); tsubokuro23@yahoo.co.jp (R.N.); nakanonosakue@yahoo.co.jp (N.H.); miyazawa-hideaki@kamata.jcho.go.jp (H.M.); itoi@tokyo-med.ac.jp (T.I.); 2 Department of Gastrointestinal and Pediatric Surgery, Tokyo Medical University, 6-7-1 Nishishinjuku Shinjuku-ku, Tokyo 160-0023, Japan; ynagakawa@gmail.com (Y.N.); akihikot@tokyo-med.ac.jp (A.T.)

**Keywords:** high-intensity focused ultrasound (HIFU), pancreatic cancer, tumor ablation, combination therapy

## Abstract

High-intensity focused ultrasound (HIFU) is a novel advanced therapy for unresectable pancreatic cancer (PC). HIFU therapy with chemotherapy is being promoted as a novel method to control local advancement by tumor ablation. We evaluated the therapeutic effects of HIFU therapy in locally advanced and metastatic PC. PC patients were treated with HIFU as an optional local therapy and systemic chemotherapy. The FEP-BY02 (Yuande Bio-Medical Engineering) HIFU device was used under ultrasound guidance. Of 176 PC patients, 89 cases were Stage III and 87 were Stage IV. The rate of complete tumor ablation was 90.3%, while that of symptom relief was 66.7%. The effectiveness on the primary lesions were as follows: complete response (CR): n = 0, partial response (PR): n = 21, stable disease (SD): n = 106, and progressive disease (PD): n = 49; the primary disease control rate was 72.2%. Eight patients underwent surgery. The median survival time (MST) after diagnosis for HIFU with chemotherapy compared to chemotherapy alone (100 patients in our hospital) was 648 vs. 288 days (*p* < 0.001). Compared with chemotherapy alone, the combination of HIFU therapy and chemotherapy demonstrated significant prolongation of prognosis. This study suggests that HIFU therapy has the potential to be a novel combination therapy for unresectable PC.

## 1. Introduction

Pancreatic cancer (PC) is one of the most severe malignant tumors. PC has been increasing worldwide in recent years, and it has become one of the most important malignant tumors that contribute mortality. The overall prognosis for PC is poor, and for all stages of PC combined, the 1-year relative survival rate is approximately 20%, while the 5-year rate is approximately 7% [1]. Many cases of PC are diagnosed at an advanced stage even with recent advances in imaging, and the unresectable situation still accounts for approximately 60% of cases. PC patients who undergo common treatments such as surgery, chemoradiotherapy, and chemotherapy still receive insufficient benefit because of their histological characteristics. They are expected to both increase antitumor efficacy and improve the clinical benefit response (CBR). However, the therapeutic effects of chemotherapy and chemoradiotherapy are not sufficient to satisfy unresectable PC. Moreover, severe pain caused by advanced PC is extremely difficult to treat and can significantly affect the quality of life of patients. Therefore, new advances in treatment are expected for unresectable PCs.

Recently, the advancement of ultrasound has been expanded to diagnostic imaging, needle guidance, biopsy for percutaneous procedures, and directive treatment of tumors. Moreover, ultrasound technology has been developed for the use of focused ultrasound energy for therapeutic purposes, such as tissue ablation. High-intensity focused ultrasound (HIFU) therapeutic systems have the ability to ablate deep tissues inside the body from an external source. The effects of HIFU can result in cell destruction and tissue necrosis. HIFU therapy has achieved some success in treating several different types of tumors in the fields of general surgery, urology, gynecology, and neurology [2,3]. HIFU is also expected to be a new, advanced, noninvasive therapy for unresectable PC [4,5,6,7,8,9,10,11,12,13,14,15,16,17,18,19,20,21,22,23,24,25,26,27,28,29,30,31,32,33,34,35,36,37,38,39,40,41,42,43,44,45,46,47,48,49,50,51,52,53,54,55,56,57,58]. HIFU therapy with chemotherapy is being promoted as a new method to control local advancement by ablation of tumor, and it mainly achieves relief of pain caused by PC. Therefore, we planned and performed a clinical safety trial of HIFU therapy for unresectable PC in our institution from December 2008 to December 2011. One report showed the safety of HIFU therapy for unresectable PC [4]. Moreover, the therapeutic effects of HIFU therapy for unresectable PC are as expected. The aim of our study was to evaluate the therapeutic effects of HIFU therapy for unresectable PC.

## 2. Results

### 2.1. General and Clinical Data

There were no significant differences in characteristics between the Stage III and Stage IV of the group treated by HIFU with chemotherapy (H Group). There were significant differences between the Stage III of gemcitabine (GEM) and the Stage IV of S-1 in the chemotherapy regimen with HIFU administration (*p* = 0.035, *p* < 0.05). There were no significant differences in the characteristics between the Stage III and Stage IV of the group treated with chemotherapy only (C Group). There were also several GEM regimens in chemotherapy regimens and no significant differences between the Stage III and Stage IV.

All pancreatic tumors were visualized using the HIFU ultrasound monitor system, and the tumor locations were fixed. They were then treated under visualization. HIFU therapy was repeated every 2–3 months when the therapeutic effect was SD (stable disease) or PD (progressive disease). The results of therapeutic data are shown in Table 1. The number of new treatment cases was 176, and the total number of treatment cases, including repeated treatment, was 332. The number of patients who received HIFU therapy only once was 93, and the number of repeated treatment cases was 83. The mean number of repeated treatments was 2.94 (2–12). The mean number of treatment sessions was 2 (1–5), the mean total therapeutic time per session was 89.4 ± 66.8 min, the mean total number of irradiation per session was 1709.6 ± 1125.7 shots, the rate of given anesthesia was 0%, and the pain-killer administration rate was 0.6%. The incidence of adverse events was 2.8%, which included two pseudocysts, one pancreatitis (mild), one skin burn (mild), and one gastric ulcer (mild). No severe adverse events were observed in this study. There were no significant differences in the therapeutic data between the two groups, except for mean treatment sessions and mean total number of irradiations.

### 2.2. Therapeutic Effect of HIFU Therapy

**Figure 1:**
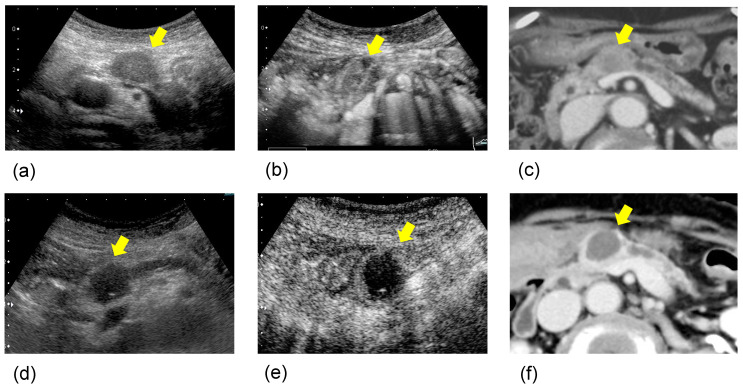
A case with a better prognosis after HIFU therapy: The patient was an 83-year-old woman with pancreatic body cancer 20 mm in size. (**a**) B-mode ultrasonography (US) (pre HIFU); the pancreatic body tumor showed hypoechogenicity in B-mode US. (**b**) Contrast-enhanced ultrasonography (CEUS) (pre HIFU); the tumor showed isovascular on CEUS. (**c**) Contrast-enhanced computed tomography (CECT) (pre HIFU); the tumor showed low-density area in CECT. (**d**) B-mode US (post HIFU); after one session of HIFU therapy, the tumor showed echogenic changes in B-mode US. (**e**) CEUS (post HIFU); the tumor showed hypovascular changes on CEUS. (**f**) CECT (post HIFU); it showed a low-density area with no contrast effect, and complete coagulative necrosis was observed. Thereafter, she survived for 2 years without active treatment.

Therapeutic effect data are shown in Table 2. Mean tumor size before and after treatment were 33.3 (±10.9) and 33.5 (±11.3), respectively. CT image was used to assess tumor size. There were no significant differences before and after HIFU therapy. The rate of complete tumor ablation was 90.3%. CBR consists of factors such as pain, appetite, fatigue, sleep, and weight. The rate of pain relief was 63.8%, and the rate of symptom relief effect was 66.7%. The effectiveness of the treatment in primary lesion was CR (complete response): 0, PR (partial response): 21, SD: 106, and PD: 49 cases. The primary disease control rate (DCR) was 72.2%. Post-treatment after HIFU therapy was operation in eight, chemotherapy in one hundred and forty-nine, arterial infusion chemotherapy in one, immuno-therapy in four, and BSC in twenty-two cases. A case with a better prognosis after treatment is shown in Figure 1. Regarding the rate of symptom relief, the rate of no worsening change in CA19-9 level, and primary DCR, Stage III was significantly better than Stage IV.

**Figure 2:**
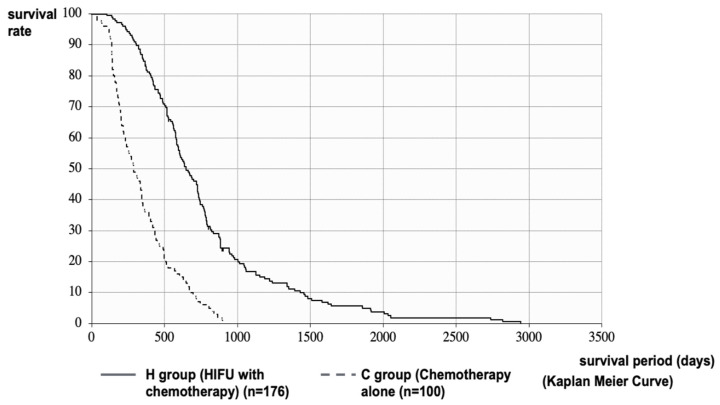
Survival curve after diagnosis of PC: MST after diagnosis of PC in the H and C Groups were 648 vs. 288 days (21.3 vs. 9.5 months), respectively. The MST of the H Group was significantly longer than that of the C Group.

The median survival time (MST) after diagnosis of PC in the H and C Groups was 648 vs. 288 days (21.3 vs. 9.5 months), respectively. The MST of the H Group was significantly longer than that of the C Group (*p* < 0.001, log-rank test; HR = 0.2758 [95%CI: 0.2103–0.3617]) (Figure 2). It was necessary to consider the effect of treatment methods before HIFU therapy and patient status in the comparison after diagnosis of PC. Therefore, we performed a stratified analysis matching the conditions of the H and C Groups. In the C Group, 89 patients (C’ Group) were stratified by excluding patients with PS ≥ 3 at the time of diagnosis who were not eligible for HIFU therapy, patients with multiple metastases or marked ascites due to peritoneal metastasis who could not be expected to have a prognosis of more than 4 weeks, patients with deep pancreatic tail cancer that could not be treated with HIFU, and patients with PC invading the gastrointestinal tract.

**Figure 3:**
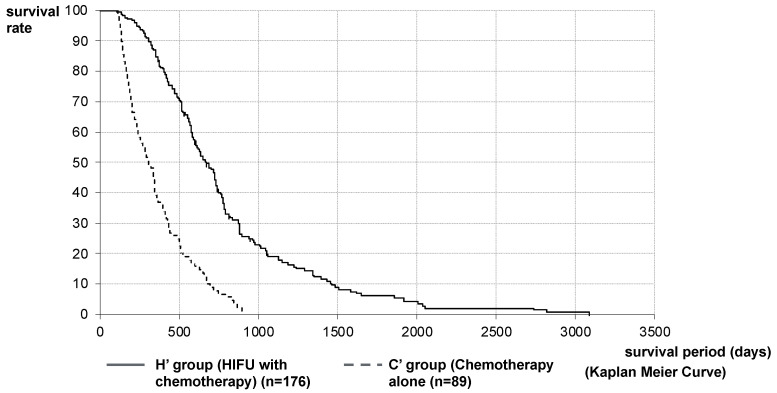
Survival curve after diagnosis of PC in Stratified Analysis 1: stratified analysis matching the conditions with H and C Groups was performed on 176 patients in the H Group (H’ Group) and 89 patients in the C Group (C’ Group). MST after diagnosis of PC in the H ‘and C’ Groups were 648 vs. 310 days (21.3 vs. 10.2 months), respectively. The MST of the H’ Group was significantly longer than that of the C’ Group.

**Figure 4:**
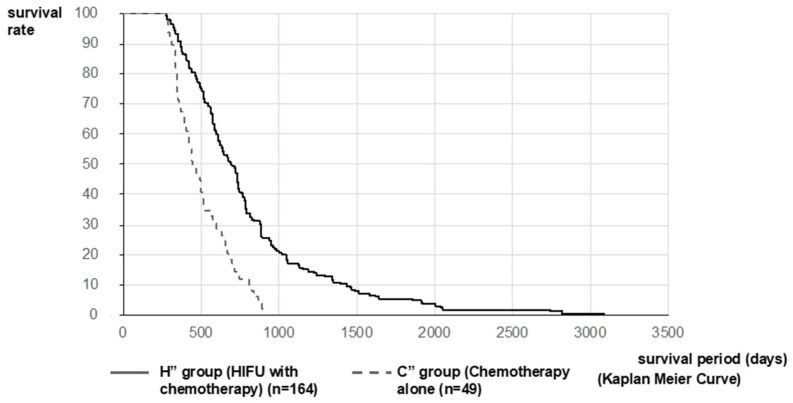
Survival curve after diagnosis of PC in Stratified Analysis 2: Considering that the mean treatment time to reach HIFU therapy was 288.5 days and that it was the third-line treatment, we compared 49 patients in C” Group, where the second-line chemotherapy was PD and 164 patients in the H” Group. MST after diagnosis of PC in the H” Group and C” Group was 733 vs. 522 days (24.1 vs. 17.2) months, respectively. The MST of the H” Group was significantly longer than that of the C” Group.

The analysis was performed on 176 patients (H’ Group) and 89 patients (C’ Group). MST after diagnosis of PC in the H’ and C’ Groups was 648 vs. 310 days (21.3 vs. 10.2 months), respectively. The MST of the H’ Group was significantly longer than that of the C’ Group (*p* < 0.001, log-rank test; HR = 0.2850 [95%CI: 0.2155–0.3768]) (Figure 3). Furthermore, considering that the mean treatment time to reach HIFU therapy was 288.5 days and that it was the 3rd-line treatment, we compared 49 patients in the chemotherapy group (C” Group), where the second-line chemotherapy was PD and able to be transferred to the third-line treatment with second-line chemotherapy patients excluding best supportive care (BSC) patients in the HIFU Group (164 patients, H” Group). MST after diagnosis of PC in the H” Group and C” Group were 733 vs. 522 days (24.1 vs. 17.2) months, respectively. The MST of the H” Group was significantly longer than that of the C” Group (*p* < 0.001, log-rank test; HR = 0.4058 [95%CI: 0.2760–0.5965]) (Figure 4).

**Figure 5:**
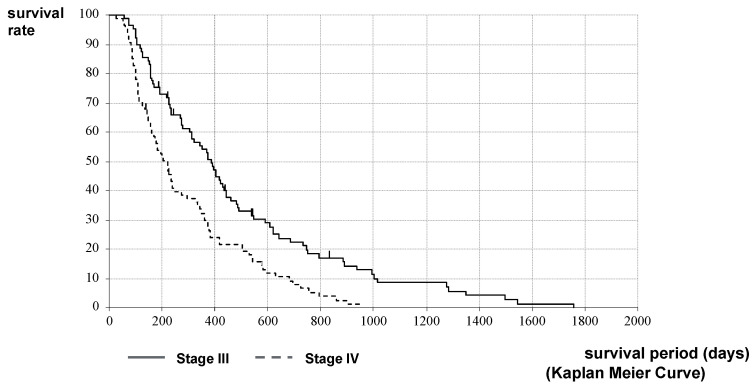
Survival curves of Stages III and IV after HIFU therapy in the H Group: A comparison of MST after HIFU therapy in Stages III and IV of the H Group was 372 vs. 220 days (12.2 vs. 7.2 months), respectively. The MST of Stage III was significantly longer than that of Stage IV.

The stage classification may be different at the time of HIFU therapy from that at the time of PC diagnosis. Therefore, the comparison of survival by stage classification was performed from the time of HIFU therapy. The MST after HIFU therapy in Stages III and IV of the H Group was 372 vs. 220 days (12.2 vs. 7.2 months), respectively. The MST of Stage III was significantly longer than that of Stage IV (*p* < 0.001, log-rank test; HR = 0.6056, 95%CI [0.4440–0.8260]) (Figure 5).

## 3. Discussion

This study showed significant results in the outcome of HIFU therapy for unresectable PC. This suggests that HIFU therapy has the potential to be a new strategy for combination therapy for unresectable PC. Furthermore, this is the first report of a long-term prospective clinical study on a novel treatment other than chemotherapy and radiotherapy for unresectable PC.

Surgical resection is the main therapy for malignant tumors in the hepatobiliary-pancreatic field; however, clinically, there are many cases in which resection is difficult due to tumor progression. In addition, surgery is a highly invasive treatment, and a patient may not be amenable to surgery depending on the general condition of the elderly and patients with high-risk comorbidities. In recent years, along with the development of medical technology, there are increasing expectations that minimally invasive local treatment will be an option for treatment, even in the hepatobiliary-pancreatic field. Among them, it is particularly expected in the area of PC due to its poor prognosis. PC is one of the worst prognoses of malignant cancers. The difficulty in treatment is due to the rapid progression and complexity of anatomical characteristics. The median survival of unresectable PC is approximately 10 months, and the therapeutic results for unresectable PC patients who undergo common treatments such as chemoradiotherapy and chemotherapy are still not satisfactory. Moreover, these patients mostly struggle severe cancer pain. Minimally invasive HIFU therapy may be useful as an optional treatment for patients with inoperable and progressive symptoms and may also become a palliative therapy for unresectable PC.

HIFU is a new method for the non-invasive treatment of PC using ultrasound. Ultrasound is a widely used diagnostic tool worldwide. It is possible to shift from the diagnosis area to the treatment area depending on the ultrasonic intensity and irradiation time [59,60]. The principle of HIFU therapy is to treat deep internal tissues from outside the body using the HIFU system. The ultrasonic waves are radiated from the transducer inside the semi-circular probe, and the vibration energy is focused on the target area, which is the center of curvature [2,7,61]. As a result, the heat is converted to 80.0–98.6 degrees depending on the absorption coefficient of the tissue. The tissue temperature reached a high temperature within seconds. HIFU has two biological actions [59,60]: thermal action (heating action) and non-thermal action (mechanical action). Mechanical effects are associated with cavitation, microstreaming, and radiant forces. These two actions cause coagulative necrosis of tissues, degeneration/apoptosis, cell destruction, and fibrosis, resulting in a therapeutic effect [2,4,7,61]. As for the relationship between tumor and depth, the greater the depth of the tumor, the greater the attenuation of the ultrasound waves during the transmission process (as the depth increases by 1 cm, the intensity is attenuated by approximately 20%). The intensity becomes very low when it reaches the target and cannot reach the expected effect. Therefore, if the target is located too deep, the intensity can be too low to induce the expected effect. In this study using the HIFU device (FEP-BY 02, Beijing Yuande Bio-Medical Engineering Co., Ltd., Beijing, China), it is only indicated for cases where the depth of the tumor is within 2–10 cm from the surface of the skin. The pancreas is a highly sensitive organ that may cause serious inflammatory changes owing to the effect of heat using HIFU. However, no serious adverse events have been reported in previous clinical and animal experimental reports [2,3,4,5,6,7,8,9,10,11,12,13,14,15,16,17,18,19,20,21,22,23,24,25,26,27,28,29,30,31,32,33,34,35,36,37,38,39,40,41,42,43,44,45,46,47,48,49,50,51,52,53,54,55,56,57,58,61,62,63,64,65,66,67,68,69,70]. We also reported on the safety of HIFU therapy in a clinical study to verify the safety of unresectable PC [4].

The outcome in this study showed the usefulness of the therapeutic effect and symptom relief effect in patients with unresectable PC, as shown in Table 2 and Figure 1, Figure 2, Figure 3 and Figure 4. The palliative effect and local controllability were significantly better in Stage III, as shown in Table 2 and Figure 5. From these results, HIFU is an effective treatment for local control, and benefits such as antitumor effect, potential for additional treatment, and symptom relief effect can be expected. It is non-invasive and has the potential to become a treatment option for unresectable PC in the future. Comparing the MST after the diagnosis of PC, the MST of the H Group was significantly better than that of the C Group. Furthermore, when the MST was compared by stratified analysis of the H and C Groups, taking into account the treatment before HIFU therapy, the duration of treatment, and the patient status, MST was also significantly prolonged in both HIFU therapy groups. This indicates that MST was significantly prolonged after HIFU therapy. In addition, the comparison between stages indicated that Stage III significantly prolonged MST after HIFU therapy. This study suggests that HIFU therapy is one of the strategies for unresectable PC, especially for locally advanced PC, and it is expected that the prognosis will be prolonged by combining HIFU therapy as local control with chemotherapy as a systemic therapy. Moreover, it is necessary to note the prolongation of Stage IV prognosis. It is speculated that HIFU therapy may activate the immune system [5]. This is the so-called abscopal effect, in which HIFU treatment of the primary tumor, even in patients with multiple metastases, exerts an antitumor effect on the metastases. It is suggested that this may have contributed to the prognosis prolonging effect, which is a subject for further study.

The potential benefits of HIFU therapy for unresectable PC include symptom relief effect, prognostic prolongation effect, anti-tumor effect, potential for re-treatment and additional treatment, the possibility of strengthening drug delivery (drug penetration) [8,14,15,16,31,40,53,54,66,67,68,69,70], and the possibility of activating the immune system [5]. In addition, HIFU has the advantage that, unlike radiation therapy, it can be used as treatment any number of times repeatedly and can be used even after radiation therapy. In this study, 47% of cases had multiple HIFU therapies every 2–3 months (mean: 2.9 times). Furthermore, there is no need to administer anesthesia or sedation. The patient can be awake during the treatment, and the treatment time is short. Beyond our current study, further researches using the various effects of HIFU in cancer treatment are needed. First, HIFU therapy has been suggested to enhance drug penetration and activate cancer-specific immunity [5]. It is also important to conduct research on the potential benefits of HIFU, such as the immunopotentiation effect, the abscopal effect, and drug (anticancer drug) penetration effect [2,4,7,54,59,60,61,62,63,64,65,66,67,68,69]. Second, it has long been known that the cauterization area of HIFU is enhanced by microbubbles (enhancement of the cavitation effect) [70]. Further progress is expected in new microbubbles (nanoparticle level) specialized for treatment, development of HIFU enhancer, drug delivery treatment by combination use of HIFU with nanomicelle anticancer agents, and ultrasonic endoscopic HIFU [71].

This study has some limitations. First, this was performed manually by a single doctor. Second, there is an element of personalized treatment according to each patient’s situation. Third, as this was a single-center study, randomized controlled trials should be conducted to determine whether HIFU therapy for unresectable PC results in local tumor control and clinically beneficial outcomes such as the prolongation of survival and symptom relief in the future. Fourth, although this was a prospective study, there is still an apparent patient selection bias based on the timing of HIFU therapy, prior therapy, and chemotherapy regimen of the patients.

## 4. Materials and Methods

### 4.1. Materials

This study was a prospective single-center study with continuous registration by non-random sampling. This trial was performed at Tokyo Medical University, Department of Gastroenterology and Hepatology in Japan from December 2008 to March 2019, supported by the Tokyo Medical University Cancer Research Foundation. All patients provided written informed consent before enrollment in the study. This clinical trial was approved by the ethics committee of our hospital (approval number: 890), registered and initiated with the University Hospital Medical Information (UMIN 000009969).

A total of 176 unresectable PC patients were included in this study; it also included 30 patients evaluated in a safety assessment [4]. According to the Union for International Cancer Control (UICC) guidelines, 176 patients were classified into 89 patients as Stage III and 87 patients as Stage IV. The details of the characteristics of the HIFU with chemotherapy group (H Group) are shown in Table 3. The sex ratio was 90 males to 86 females. The mean age was 64 (range: 22–92) years. Performance status (PS) was 0–2 (PS: 0, 85; PS:1, 87; and PS:2, 4 cases). The tumor was located at the head of the pancreas in 49 cases, at the uncus in 21 cases, body in 73 cases, body-tail in 7 cases, tail in 4 cases, and others (recurrence region) in 22 cases. The mean tumor size before HIFU therapy was 33.3 ± 10.9 mm.

The details of treatment before HIFU therapy (with overlap) were chemotherapy in 97, chemoradiotherapy in 42, surgical resection in 22, BSC in 12, immunotherapy in 8, arterial infusion chemotherapy in 5, and irreversible electroporation (IRE) in 3 cases. The mean duration from diagnosis to HIFU therapy was 288.5 (range: 3–2472 days). The details of the chemotherapy regimen for HIFU administration are shown in Table 3 There were many GEM regimen for chemotherapy during HIFU therapy. Since the GEM and/or S-1 regimen was approved in Japan at the start of HIFU therapy, many patients received these menus. FOLFIRINOX therapy was approved at the end of 2013, and GEM + nab-PTX therapy was approved by the end of 2014. Several patients were initially treated with these new regimens and finally returned to the GEM single-agent regimen before receiving HIFU therapy.

The characteristics of patients and details of the chemotherapy regimen in HIFU administration in the H Group are shown. There were many GEM regimens for chemotherapy with HIFU administration. There are 11 patients who did not receive chemotherapy at the time of HIFU administration, but resumed chemotherapy immediately after HIFU therapy. The details were 6 patients in Stage III (GEM; 3, S-1; 3) and 5 patients in Stage IV (GEM; 2, S-1; 3).

#### 4.1.1. Inclusion Criteria of the H Group

Patients with an unresectable progressive PC, where the usual therapy is not indicated, ineffective, and/or the pain control was defective, were included. Patients are selected based on the following criteria: (1) age ≥ 20 years; (2) history of previous therapy was not required; (3) if there was a history of radiological therapy, the patient must have a period of no treatment for 4 weeks or more; (4) for a history of other therapies, the period it judged not to influence this study is open; (5) the function of the main internal organs (heart, liver, lung, and kidney) was maintained; (6) WBC ≥ 2000/μL, PLT ≥ 5 × 10^4^/μL; (7) a survival of 4 weeks or more could be anticipated; (8) an applicable lesion where visualization was possible using US; and (9) the route of the focus of ultrasonic waves could be secured, where the depth of the tumor was within 2–10 cm from the skin.

#### 4.1.2. Exclusion Criteria of the H Group

Patients were excluded if they qualified for any one of the following criteria: (1) if there was a presence or a possibility of having a malignant tumor on other internal organs; (2) if with pregnancy or the possibility of conceiving; (3) cases in which active inflammation and infection are complicated; (4) cases which had obstructive jaundice—however, these become acceptable after drainage; (5) patient had serious cardiopathy and encephalopathy; (6) possibilities of stanching difficulty was expected in cases such as antiplatelet therapy and anticoagulant therapy; (7) patient deemed by the investigator as an inappropriate subject for this research; (8) the lesion was not visualizable on US; and (9) the depth of the tumor was more than 10 cm from the skin or other ultrasound focusing route was not available.

One hundred patients with PC who received chemotherapy at the same time at our hospital were included in this study (C Group; chemotherapy alone). The subjects were patients with histologically diagnosed PC. The details of patient characteristics are presented in Table 4 The sex ratio was 57 males and 43 females. The mean age was 63.0 (range: 45–75) years. PS was 0–3 (PS: 0; 35, PS: 1; 31, and PS: 2; 23, PS: 3; 11 cases). The tumor was located at the head of the pancreas in 46 cases, at the uncus in 7 cases, body in 27 cases, body-tail in 5 cases, tail in 14 cases, and others (recurrence region) in 1 case. The mean tumor size before chemotherapy was 34.2 ± 9.7 mm. The details of the chemotherapy regimens are shown in Table 4 Many GEM regimens were administered for chemotherapy. Since the GEM and/or S-1 regimen was approved in Japan at the start of HIFU therapy, many patients received this regimen.

The characteristics of the patients and details of the chemotherapy regimen of the C Group are shown. There were also several GEM regimens in Group C.

#### 4.1.3. Inclusion Criteria of the C Group

Patients with histologically diagnosed pancreatic cancer were included in the study if they fulfilled the following criteria: (1) age ≥20, ≤75 years old; (2) performance status 0–3; (3) no heterogeneous or concurrent multiple cancers; (4) patients with or without treatment in the previous 2 weeks; (5) patients with major organs intact, Hb ≥ 8.0 g/dL, WBC ≥ 4000/μL, Neutrophil ≥ 2000/μL, PLT 10 × 10^4^/μL, ALT and AST less than 2.5 times the upper limit of the institutional standard, T-bil ≤ 2.0 mg/dL, Cre ≤ 1.5 mg/dL, Ccr ≥ 60 mL/min; (6) for patients with obstructive jaundice or liver metastases, the upper limit of 5 times or less the institutional normal value should be confirmed before administration of treatment; (7) for patients with obstructive jaundice, the upper limit of the institutional normal value being 2.5 times or less after reducing the yellowing level should be confirmed before drug administration; (8) patients who have no serious complications that make administration inappropriate; (9) patients who are capable of oral intake; (10) patients who are expected to survive for at least 4 weeks; and (11) patients who have given written consent to participate in this study for themselves and their families.

#### 4.1.4. Exclusion Criteria of the C Group

Patients are excluded if they qualify for any of the criteria: (1) if interstitial pneumonia or pulmonary fibrosis is present; (2) patients with difficult-to-control diabetes mellitus, hepatic impairment, angina pectoris, or myocardial infarction within 3 months of onset; (3) patients with severe infectious diseases; (4) pregnant women, lactating women, and women who may have or intended to become pregnant; (5) patients with severe drug allergy; (6) patients with serious complications; and (7) patients who were judged by the investigator to be inappropriate for the safe conduct of this study.

### 4.2. Methods

The patients who participated in this research were contacted by the diagnostic imaging committee. We evaluated the imaging data and confirmed the adaptation. Once approved for research adaptation, the patient was given a registration number in order to participate in the study. PC patients in the H Group were treated with a combination of local therapy with HIFU and systemic chemotherapy. The treatment was aimed at complete ablation of the tumor. The treatment time was approximately 30 min to 1 h every session from the viewpoint of accident prevention and tumor size; multiple treatments within 1 week were defined as one session. HIFU therapy was repeated every 2–3 months when the therapeutic response was SD or progressive disease PD according to the Response Evaluation Criteria in Solid Tumors (RECIST) guidelines; these were classified as stable or progressive disease after evaluation of the images, tumor markers, and symptoms. Chemotherapy in Group C was administered according to the usual method after diagnosis.

### 4.3. Devices

**Figure 6:**
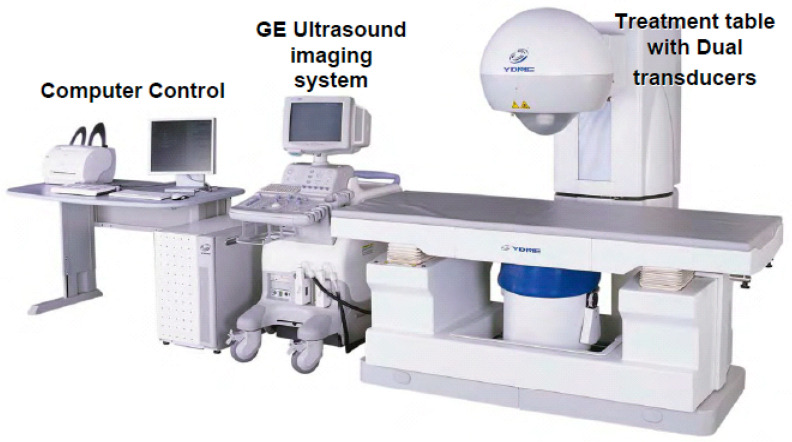
FEP-BY 02 Series HIFU Therapy System: the HIFU therapeutic system used was the Model FEP-BY02 HIFU system. The HIFU system includes a computer control, GE ultrasound imaging system, and a treatment table with dual transducers (upper and lower sides). The therapy for PC made use of an upper transducer.

**Figure 7:**
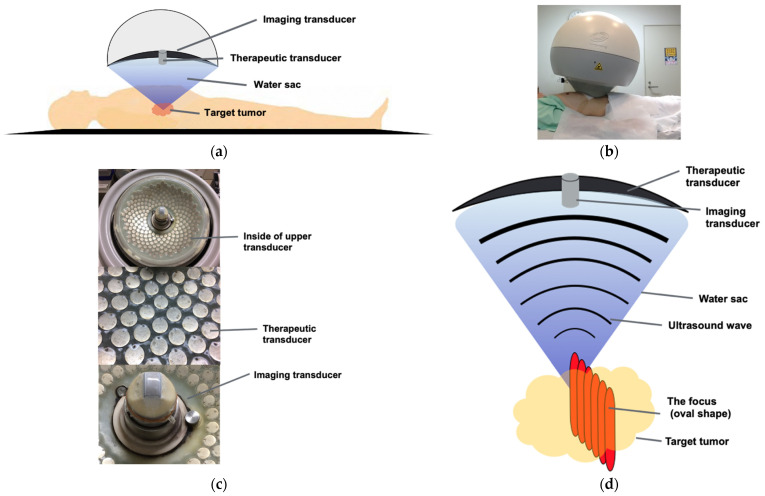
The principles of HIFU therapy system: (**a**,**b**) This system had the capacity to deliver HIFU from an external source deep into tissues, with a large convergence angle. The aperture diameter was 37 cm, the radius of curvature was 25.5 cm, and the therapeutic transducer had a 251 element array. The configuration (aperture diameter and radius of curvature) is used to reduce the risk of skin burn. The upper transducer was accompanied by a water sac filled with degassed water. The abdomen was pressed by the water sac, and the target was visualized with ultrasonic waves for observation. A total of 251 ultrasonic waves embedded in the transducer were focused on the target through the water sac. (**c**) Upper transducer; upper transducer with the imaging probe and therapeutic transducer with 251 elements array (frequency: 1.1 MHz). (**d**) Targeting system: The ultrasound wave converges to a focus point. The multi-element array technology ensures an even acoustic field and an 80° angle of convergence. The focus was an oval shape, and the practical focused volume was approximately 3 × 3 × 10 mm, whereas the effective focused volume was 6 × 6 × 10 mm. The target tumor was treated by shifting every 3 mm while seeing in real-time US.

The HIFU therapeutic system used was the Model FEP-BY02 HIFU system supplied by Beijing Yuande Bio-Medical Engineering Co., Ltd. (Beijing, China) (Figure 6). The FEP-BY Series HIFU system has the capacity to deliver high-intensity focused ultrasound from an external source deep into tissues, with a large convergence angle (Figure 7). The special characteristic feature of this device is that it possesses transducers on the upper and lower sides. The upper transducer is especially useful for the treatment of abdominal organs, particularly the pancreas. The position, size, and relationship of the tumor to the adjacent organ were determined through imaging ultrasonography before the therapy. The intestinal cavity was then cleaned by fasting. During therapy, the patients lie on their backs on a treatment table. A B-mode ultrasound scanner monitoring system was used to define the target area, treatment range, treatment layers, and power. In this study, the upper transducer was used and the treatment probe was moved according to the planned procedure. No anesthesia was required during the entire treatment procedure.

Primary parameters for treatment include: (1) input electric power of 400–1100 W; (2) unit transmit time (t1): 0.1–0.2 s; (3) intermission time (t2): 0.2–0.4 s, t2/t1 = 2/1; (4) the total shots at each treatment point were 1–10 times. The parameters should be adjusted based on the location and depth of the tumor, density of tumor tissue, and the decay rate of the ultrasound. The starting point was 300 W from the deepest part of the tumor, and the intensity was gradually increased to set the treatment intensity, taking into account the symptoms and internal changes in the echoes. At the same depth, the intensity was not adjusted, but when the tumor became shallower by about 1 cm, the focal intensity was adjusted to reduce by 50–100 W according to the symptoms. The entire treatment procedure and shift of focus were manually controlled by the computer. The temperature of the tissue of the target site would rise accordingly, and the output (between 400 and 900 W) generated to facilitate necrosis of cancer cells was confirmed by the cavitation change of the ultrasonic image during treatment. The treatment was performed by moving the transducer back and forth, left and right, and up and down by approximately 3 mm.

### 4.4. Definition

We established an effect and safety evaluation decision committee and a diagnostic imaging committee for the justice of the evaluation decision and unification. Evaluation of efficacy every 2 months was based on WHO criteria, and the response to the treatment was classified using the RECIST guidelines as complete response (CR), partial response (PR), stable disease (SD), and progressive disease (PD). Pain was evaluated using the numeric rating scale (NRS) every 2 months.

### 4.5. Outcome

The primary study endpoints were to evaluate the therapeutic effect of HIFU therapy for unresectable PC and establish a HIFU therapeutic method for PC. We evaluated the following points: (1) therapeutic effects: changes in the tumor marker (serum CA19-9 level) and evaluation of imaging: transabdominal B-mode US, CEUS, CECT, magnetic resonance imaging (MRI), and positron emission tomography (PET), mainly CECT imaging. CECT was performed and evaluated in three dynamic phases (arterial, portal, and venous phases). CEUS was performed after intravenous injection of the contrast agent Sonazoid (0.015 mL/kg body weight), wherein the arterial phase (15–20 s) and the venous phase (30–45 s) were evaluated; (2) CBR: changes in clinical symptoms: pain (NRS score: deemed effective in cases of more than 30% in pain relief), appetite, fatigue, sleep, and weight; and (3) adverse event: after the procedures, the results, such as the symptoms, blood tests, and adverse events on the following days and 1 week, were recorded. All patients were hospitalized for HIFU therapy and were observed for 1 week. The evaluations after HIFU therapy were repeated every 2 months. Furthermore, we compared the prognosis between the combination group of the H Group (n = 176 cases) and the concurrent C Group at the same period in our hospital (n = 100 cases).

### 4.6. Statistical Analysis

The Chi-square test was used for comparison between the groups. The overall survival times were calculated using the Kaplan–Meier method (Kaplan and Meier, 1958). The log-rank test was used to determine the significance of the associations. A *p* value of less than 0.05 indicated a statistically significant difference.

## 5. Conclusions

This study demonstrated the results of a long-term prospective study of HIFU therapy for unresectable PC in a large number of patients for more than 10 years. This is the first report of a long-term clinical study of a novel treatment other than chemotherapy for unresectable PC. HIFU therapy for PC has been proven to be a non-invasive treatment, and it was extremely revolutionary method that the tumor could be treated in real time with detailed imaging of the tumor by compressing the abdomen with an upper transducer in the supine position. In terms of therapeutic effects, 63.8% of the patients showed symptom relief, 72.2% showed primary disease control, and 12% showed tumor reduction. In addition, the combination of HIFU therapy and chemotherapy compared with chemotherapy alone resulted in a significant prolongation of prognosis. In a comparison of Stages III and IV, both groups showed prolonged prognosis, with Stage III having a significantly longer prognosis than Stage IV. In conclusion, the clinical application of HIFU therapy for unresectable PC has many possibilities and benefits such as antitumor effect, possibility of retreatment/additional therapy, prolongation of survival, and symptom relief. HIFU therapy combined with chemotherapy as systemic therapy could be a strategy for locally advanced unresectable PC.

## Figures and Tables

**Table 1:** General data of using HIFU and chemotherapy.

	Total	Stage III	Stage IV	*p* Value
Total number of treatment cases	332	158	174	N/A
Number of new cases	176	89	87	N/A
Number of only one time of treatment cases	93	44	49	*p* = 0.360
Number of repeated treatment cases	83	45	38	*p* = 0.360
Mean number of repeated treatments	2.94 (2–12)	3.01 (2–12)	3.32 (2–9)	*p* = 0.293
Mean treatment sessions (times)	2.0 (1–5)	2.2 (1–5)	1.9 (1–4)	*p* = 0.026 ^#^
Mean total treatment time (min)	89.4 (15–600)	96.3 (15–280)	82.2 (15–600)	*p* = 0.162
Mean total number of irradiation (shots/1 session)	1709.6 (280–6420)	1905 (280–6420)	1510 (280–4104)	*p* = 0.018 ^#^
Rate of given anesthesia (sedation) (%)	0 (0/176)	0 (0/89)	0 (0/87)	N/A
Rate of given pain-killer (%)	0.6 (1/176)	0 (0/89)	1.1 (1/87)	*p* = 0.310
Adverse event (%)	2.8 (5/176)	2.2 (2 */89)	3.4 (3 **/87)	*p* = 0.632

* Pseudocyst, Skin burn (mild), ** Pseudocyst, Pancreatitis, Gastric ulcer (mild), ^#^ *p* < 0.05.

**Table 2:** Therapeutic effect data of HIFU treatment.

	Total	Stage III	Stage IV	*p* Value
Mean tumor size; before therapy	33.3 (±10.9)	33.0 (±10.2)	33.5 (±11.6)	*p* = 0.738
after therapy	33.5 (±11.3)	33.5 (±11.3)	34.0 (±12.2)	*p* = 0.790
The rate of complete tumor ablation (%)	90.3	92.1	88.5	*p* = 0.469
The rate of symptom relief effect (%)	66.7	64.4	44.9	*p* = 0.029 *
Rate of no worsening change of CA19-9 level (%)	48.1	63.2	34.9	*p* = 0.011 *
The effectiveness of primary lesion (CR/PR/SD/PD)	0/21/106/49	0/12/60/17	0/9/46/32	N/A
Primary disease control rate (DCR) (%)	72.2	80.9	63.2	*p* = 0.009 *
Therapy after HIFU (operation/chemotherapy/arterial infusion chemotherapy/ radiation/immunotherapy/BSC)	8/149/1/0/4/22	7/74/0/0/1/12	1/75/1/0/3/10	N/A

* *p* < 0.05.

**Table 3:** The characteristics of patients (H Group; HIFU with chemotherapy).

	Total (n = 176)	Stage III (n = 89)	Stage IV (n = 87)
Age (range)	64 (22–92)	66 (22–92)	63 (28–87)
Sex (male/female)	90/86	41/48	49/38
Performance status (0/1/2/3/4)	85/87/4/0/0	49/39/1/0/0	36/48/3/0/0
Location			
(head/uncus/body/	49/21/73/	28/15/39/	21/6/34/
body-tail/tail/others *)	7/4/22	6/1/0	1/3/22
Chemotherapy regimen	(n = 165) (%)	(n = 83) (%)	(n = 82) (%)
GEM	90 (54.5)	52 (62.7)	38 (46.3)
S-1	28 (17.0)	9 (10.8)	19 (23.2)
GEM + S-1	20 (12.1)	11 (13.3)	9 (11.0)
GEM + nab-PTX	22 (13.3)	9 (10.8)	13 (15.9)
m FOLFIRINOX	5 (3.0)	2 (2.4)	3 (3.7)

* Recurrence lesion (including LN).

**Table 4:** The characteristics of patients (C Group; chemotherapy alone).

	Total (n = 100)	Stage III (n = 49)	Stage IV (n = 51)
Age (range)	63 (45–75)	63 (45–74)	64 (46–75)
Sex (male/female)	57/43	27/25	30/18
Performance status (0/1/2/3/4)	35/31/23/11/0	20/18/11/3/0	15/13/12/8/0
Location			
(head/uncus/body/	46/7/27/	22/3/17/	24/4/10/
body-tail/tail/others *)	5/14/1	2/5/0	3/9/1
Chemotherapy regimen	(n = 100) (%)	(n = 49) (%)	(n = 51) (%)
GEM	66 (66.0)	36 (73.5)	30 (58.8)
S-1	10 (10.0)	5 (10.2)	5 (9.8)
GEM + S-1	18 (19.0)	7 (14.3)	11 (21.5)
GEM + erlotinib	1 (1.0)	0 (0)	1 (2.0)
GEM + nab-PTX	4 (4.0)	1 (2.0)	3 (5.9)
m FOLFIRINOX	1 (1.0)	0 (0)	1 (2.0)

* recurrence lesion (including LN).

## Data Availability

The data presented in this study are available upon request from the corresponding author. The data are not publicly available, as third-party allocation of patient data is prohibited by current regulations.

## Abbreviations

Pancreatic cancer, PC; clinical benefit response, CBR; high-intensity focused ultrasound, HIFU; union for international cancer control, UICC; performance status, PS; best supportive care, BSC; irreversible electroporation, IRE; gemcitabine, GEM; albumin-bound paclitaxel, nab-PTX; University Hospital Medical Information, UMIN, inferior vena cava; IVC, response evaluation criteria in solid tumors, RECIST; complete response, CR; partial response, PR; stable disease, SD; progressive disease, PD; numeric rating scale, NRS; ultrasonography, US; contrast-enhanced ultrasonography, CEUS; computed tomography, CT; contrast-enhanced computed tomography; CECT; magnetic resonance imaging, MRI; positron emission tomography, PET; disease control rate, DCR; median survival time, MST; three-dimensional, 3D; artificial intelligence.
